# Supplementary material for: CX-5461 activates the DNA damage response and demonstrates therapeutic efficacy in high-grade serous ovarian cancer
Source: Nat Commun. 2020 May 26;11:2641. doi: 10.1038/s41467-020-16393-4 (PMC7251123; doi:10.1038/s41467-020-16393-4)
Supplement: Supplementary file 1 — Supplementary Information [file 41467_2020_16393_MOESM1_ESM.pdf]

Supplementary Information

Manuscript title: **CX-5461 Activates the DNA Damage Response and demonstrates therapeutic efficacy in High-Grade Serous Ovarian Cancer**

Sanij et al.,

Supplementary Figure 1

BRCA1-Mutated_UP GSEA

CX-5461 resistant   CX-5461 sensitive

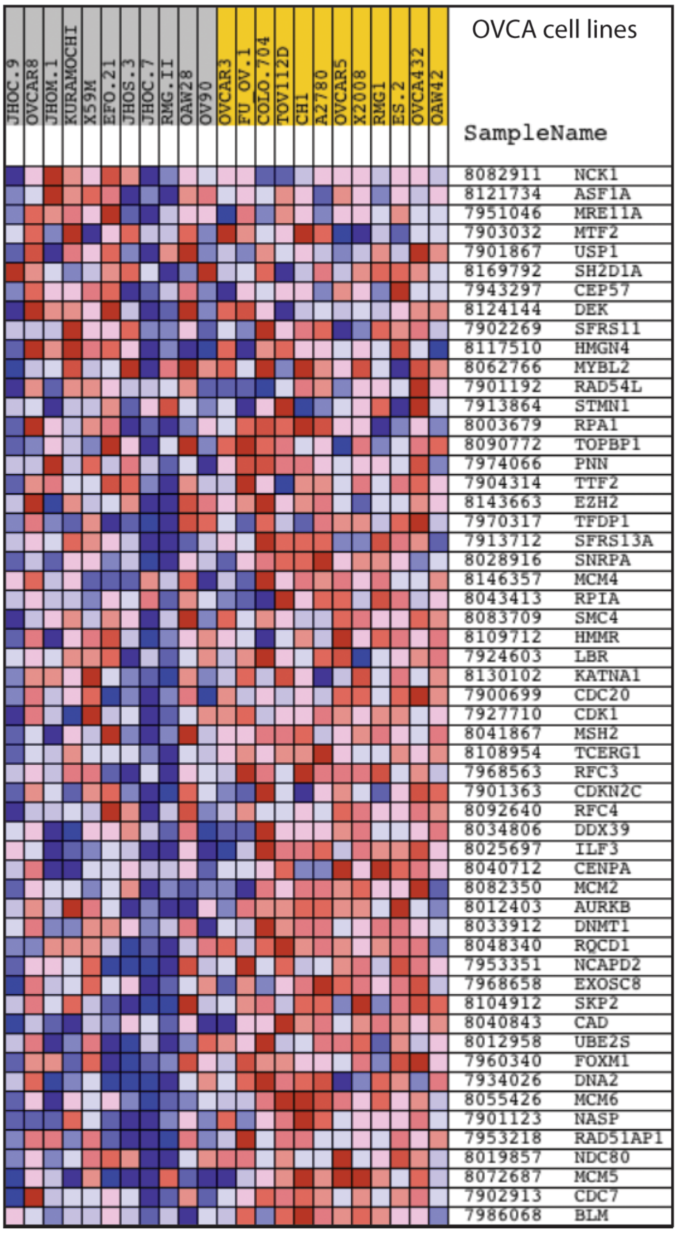

MYC_Oncogenic_Signature_UP GSEA

CX-5461 resistant   CX-5461 sensitive

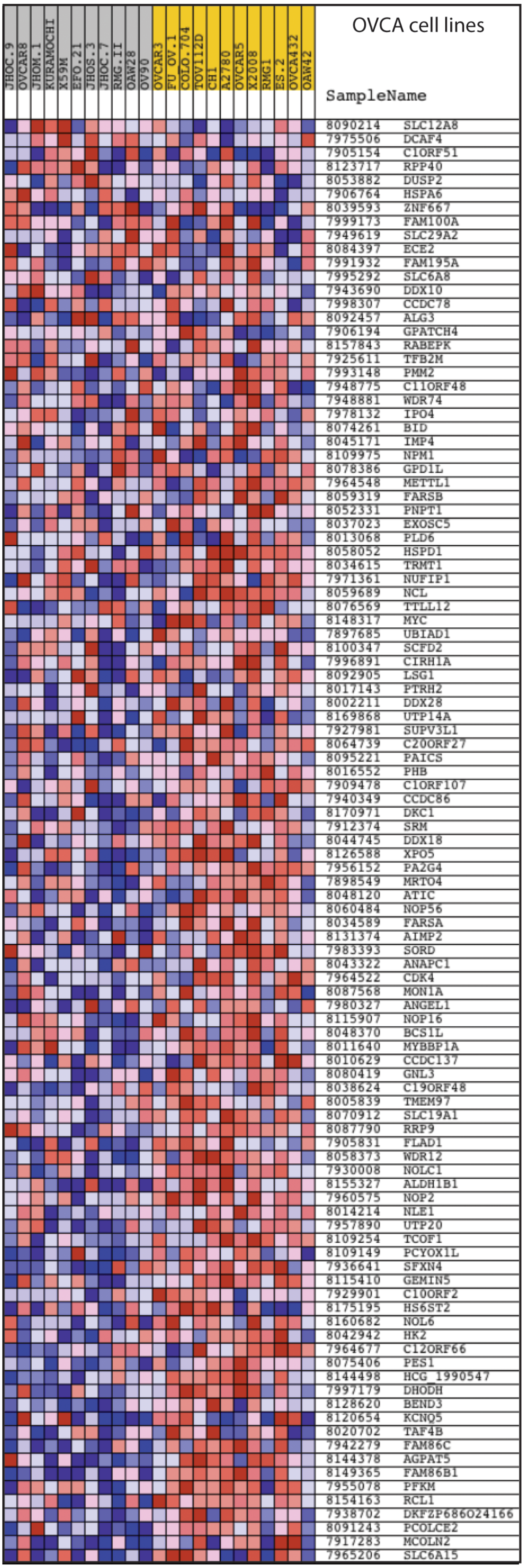

Supplementary Figure 1

Gene set enrichment analysis of microarray expression data of 12 CX-5461-sensitive and 11 -resistant cell lines. The analysis identified that the BRCA1-mutated and induced MYC targets GSEA gene sets to be enriched in CX-5461-sensitive OVCA cell lines. The heatmaps demonstrate relatively high (red) or low (blue) gene expression in the indicated sample. The gene lists are provided in Supplementary Data 2&3.

Supplementary Figure 2

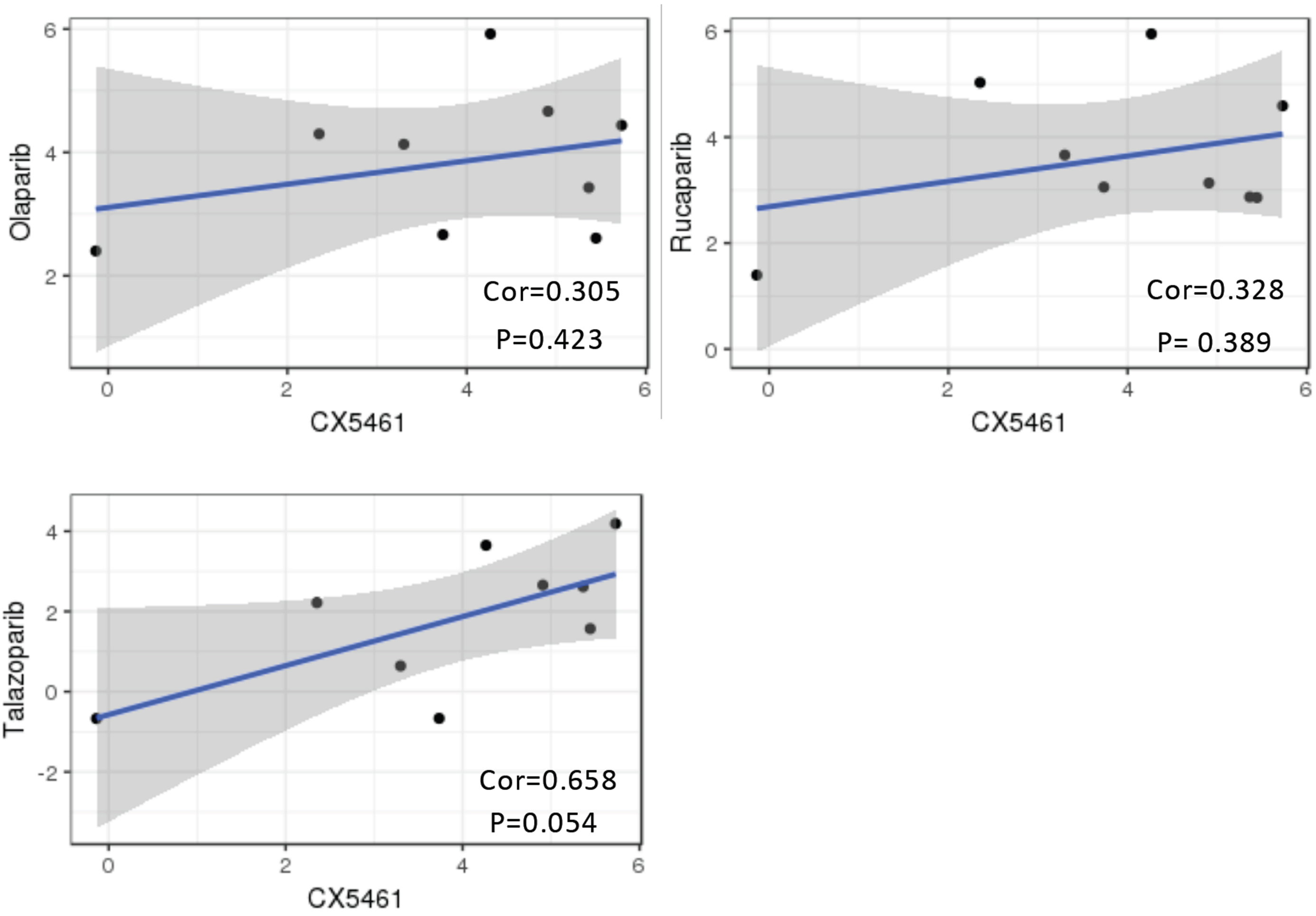

Supplementary Figure 2

Correlation between drug sensitivity measurements of CX-5461 and various PARPi in OVCA cell lines obtained from the Genomics of Drug Sensitivity database. Pearson correlation was used to measure correlation between the IC_{50} values. Two-sided test. Although a correlation between the sensitivity profiles of CX-5461 and PARPi ranging from 0.305 to 0.658 was observed, this was not significant.

Supplementary Figure 3

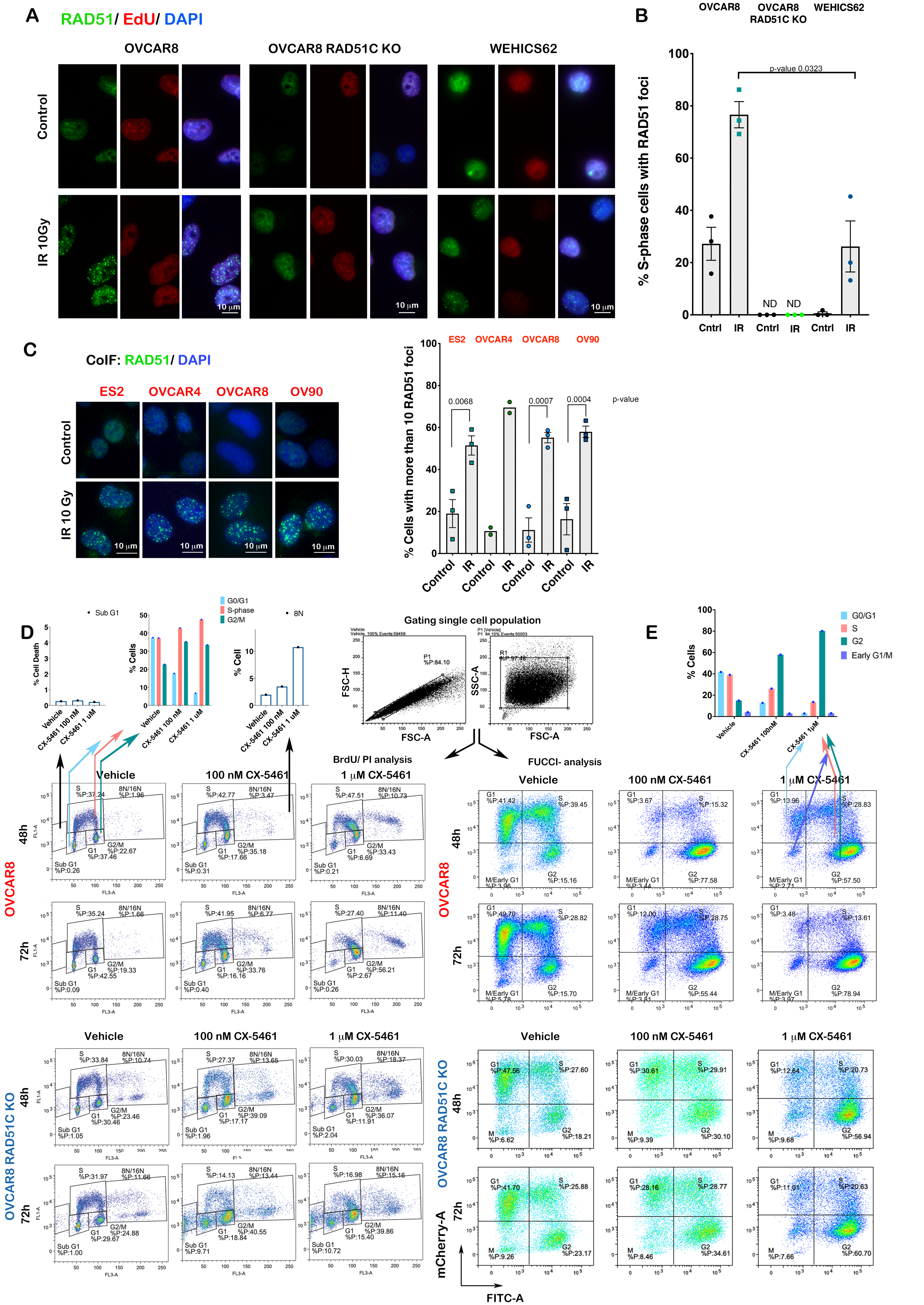

Supplementary Figure 3. A) Assessment of HR proficiency in HGSOC cell lines as reported in Kondrashova et al., Nature Communications 2018. We have re-used the data under the Creative Commons Attribution 4.0 International License [http://creativecommons.org/licenses/by/4.0/]. RAD51 foci formation was assessed 6 hours post exposure to 10 Gy ionizing radiation (IR) in HGSOC cell line OVCAR8, OVCAR8 derivative with RAD51C KO, and patient-derived HGSOC cell line with homozygous *BRCA1* hypermethylation (WEHICS62). Cells were treated with 10 μM EdU then irradiated and incubated for 6 hours. Cells were fixed with 4% paraformaldehyde and immunofluorescence for RAD51 was performed. Cells were incubated for 30 minutes at room temperature in Click-IT reaction (100 mM Tris pH 8.5, 10 nM Alexa Fluor 647-azide (Invitrogen, A10277,), 1mM CuSO4 and 100 mM Ascorbic Acid) then washed with PBS and counterstained with DAPI. Representative images of three biological replicates. **B)** Quantification of S-phase (EdU-positive) cells exhibiting > 10 RAD51 foci per cell. *n*=170 EdU-positive cells were counted per condition over three independent experiments. Mean ± SEM. Statistical analysis was performed using a two-sided student t test. ND, denotes foci not detected. **C)** RAD51 foci formation assessed 6 hours post exposure to 10 Gy IR n OVCA cell lines. Quantification of cells exhibiting > 10 RAD51 foci per cell (right panel). *n*=250 cells were counted per condition over two biologically independent experiments for OVCAR4 and three independent experiments for all other cell lines, mean ±SEM. Statistical analysis was performed using a two-sided one-way ANOVA, Tukey's multiple comparisons test (Adjusted p-values are shown). **D)** Analytical cell cycle analysis of BrdU incorporation as a function of DNA content using FACS. Cells were treated with vehicle, 100 nM or 1μM CX-5461 for 48 hours and 72 hours and labelled with BrdU for 30 minutes prior to harvest. The boxes represent S-phase BrdU-labelled populations, G0/G1, G2M and Sub G0/G1 cell populations as well as cells with > 4n DNA content. The gating strategy for quantitating % cell populations is provided in the top panel. Representative of *n*=3. **E)** FUCCI-labelled cells were treated with vehicle, 100 nM or 1 μM CX-5461 for 48 hours and 72 hours. Representative flow cytometry profile is shown of *n*=3. Each quadrant represents G0/G1, S-phase, G2, and M populations as marked on the profile and gating strategy for quantitating % cell population is provided in the top panel.

Supplementary Figure 4

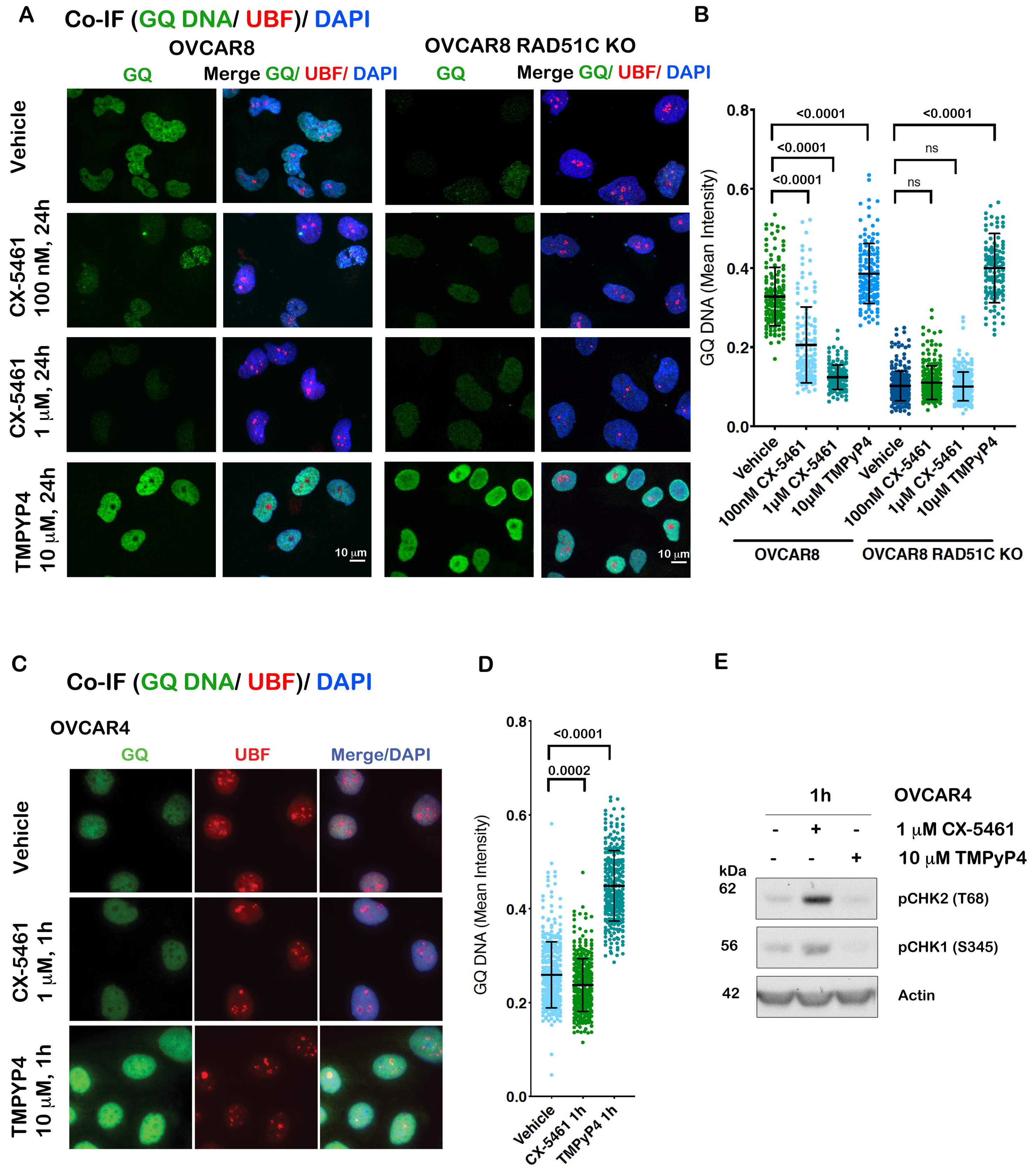

Supplementary Figure 4

CX-5461 does not stabilise GQ DNA structures in HGSOC cell lines. **A)** Co-immunofluorescence (Co-IF) of GQ DNA and UBF as a nucleolar marker in OVCAR8 and OVCAR8 RAD51C KO cells treated with either vehicle, 100 nM CX-5461, 1 μM CX-5461 or 10 μM TMPyP4 for 24 hours. Representative images of three biological replicates. **B)** Quantitation of GQ DNA immunofluorescence. Signal intensities were analyzed using Cell Profiler. *n* =75 cells per treatment condition examined over three biologically independent experiments. Error bars represent mean ± SD. **C-D)** Co-IF of GQ DNA and UBF in OVCAR4 cells, treated as indicated. Signal intensities were analyzed using Cell Profiler and normalized to corresponding vehicle controls. Representative images of three biological replicates. *n*=240 cells per treatment condition examined over three independent experiments. Error bars represent mean ± SD. Statistical analyses of difference in GQ signal intensity in **B** and **D** were performed using a two-sided Mann-Whitney t test (p-values are shown). NS: non-significant *p*-value (p-value > 0.05). **E)** CX-5461, but not TMPyP4, induces DDR. Cells were treated with vehicle, 1μM CX-5461 or 10μm TMPyP4 for 1 hour. Total protein lysates were analyzed by western blotting. Representative blots of *n* =3 biologically independent experiments. Blots shown are of samples derived from the same experiment and were processed in parallel. Actin loading controls were processed by re-probing the blots. Full scans of immunoblots are provided in Supplementary Figure 10.

Supplementary Figure 5

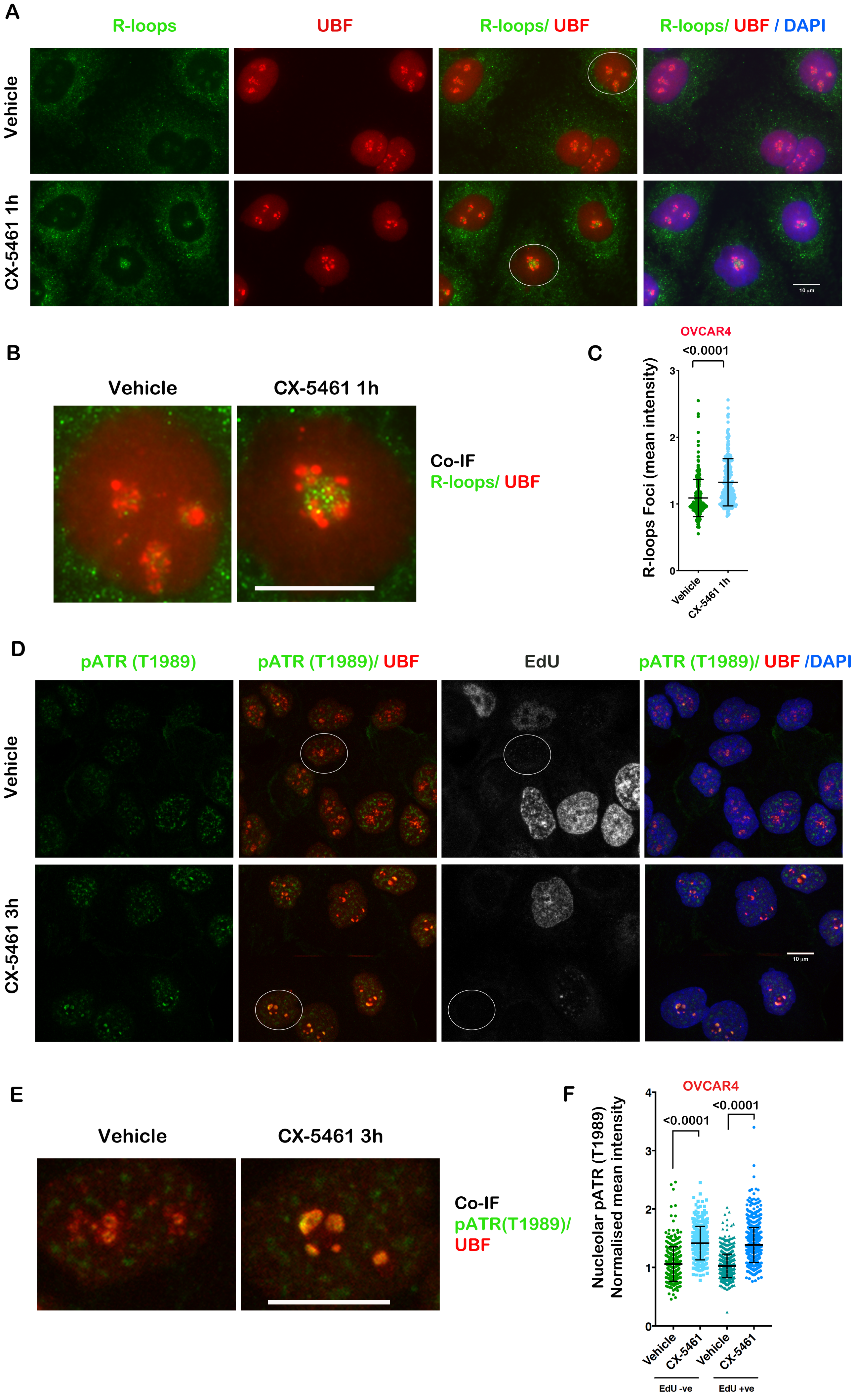

Supplementary Figure 5

A) Co-IF analysis of R-loops and UBF in OVCAR4 cells treated with vehicle or 1 μM CX-5461 for 1 hour. Representative images of three biological replicates. Cells emphasized by white circles are enlarged in (**B)**. Scale bar denotes 10 μm. **C)** Quantitation of R-loops signal intensity observed in assays of panel **A,** was performed using Cell Profiler and normalized to the median of vehicle treated controls. *n* =260 cells per treatment condition examined over three independent experiments. Error bars represent mean ± SD. Statistical analysis was performed using a two sided Mann-Whitney t test (approximate p-value is shown). **D)** Co-IF analysis of pATR (T1989) and UBF in OVCAR4 cells labelled with EdU and treated with vehicle or 1 μM CX-5461 for 3 hours. Representative images of three biological replicates. EdU negative cells emphasized by white circles are enlarged in (**E)**. Scale bar denotes 10 μm. **F)** Quantitation of signal intensity of the colocalized regions was performed using Cell Profiler and normalized to the median of vehicle treated controls. *n* =229 EdU positive and n=59 EdU negative cells per treatment condition were examined over three independent experiments. Error bars represent mean ± SD. Statistical analysis was performed using a two-sided one-way ANOVA, Krsukal-Wallis multiple comparisons test (adjusted approximate p-values are shown).

Supplementary Figure 6

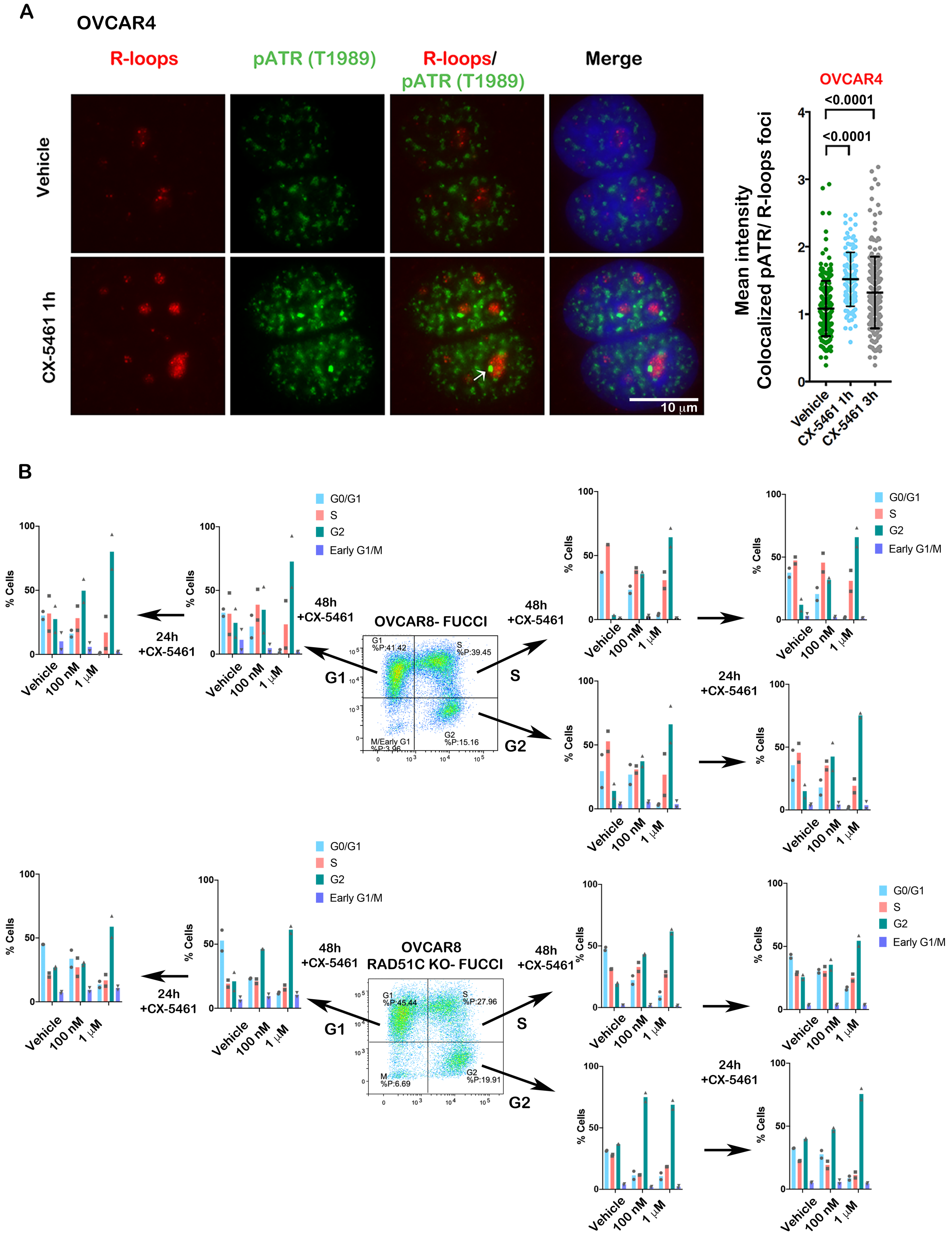

Supplementary Figure 6

CX-5461 leads to R-loops stabilisation, activation of ATR and a G2/M cell cycle arrest. **A)** Co-IF analysis of R-loops and pATR (T1989) in OVCAR4 cells treated with vehicle or 1 μM CX-5461 for 1 hour (representative images from one experiment) and 3 hours (two independent experiments). *n*=100 cells per condition were analysed using Cell Profiler. Signal intensity was normalized to median vehicle control. Error bars represent mean ± SD. Statistical analysis was performed using a two-sided one-way ANOVA, Kruskal-Wallis multiple comparison test (adjusted approximate p-values are shown). **B)** FUCCI-labelled cells were sorted in G1, S and G2 populations using BD FACSAria Fusion 4 (BD Medical Technology) before being treated with vehicle, 100 nM or 1 μM CX-5461 for 48 hours and 72 hours. Quantitation of cell cycle profiles using the FUCCI system following CX-5461 treatment are shown. Error bars represent mean ± SD of *n*=2.

Supplementary Figure 7

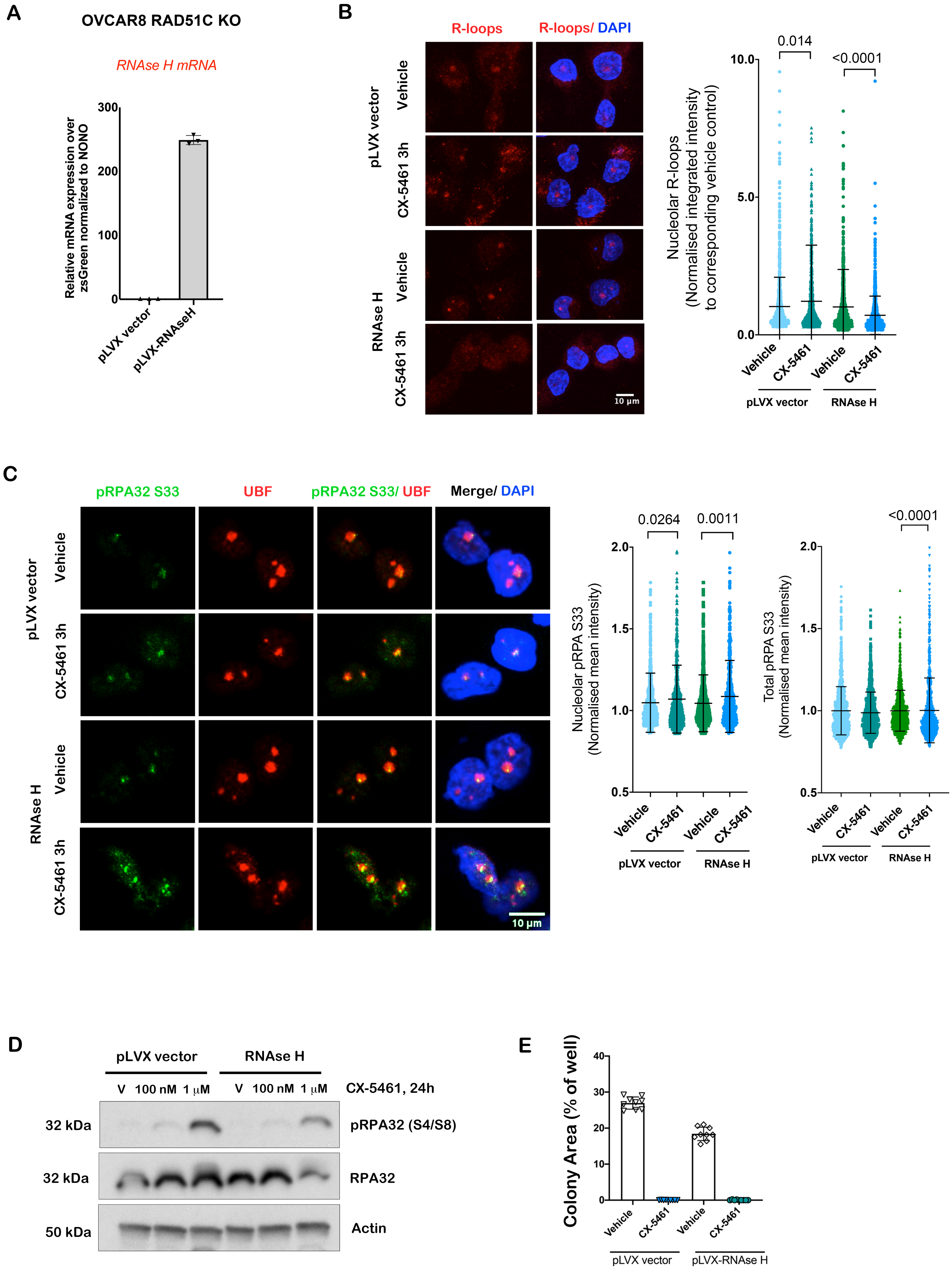

Supplementary Figure 7

R-loops stabilization contributes to CX-5461-mediated DDR but is not essential for CX-5461 efficacy **A)** OVCAR8 RAD51C KO cells were stably transduced with ribonuclease H 1 (RNase H) construct (kindly provided by Dr. Sonia Guil, Josep Carreras Leukaemia Research Institute, Barcelona, Spain). RNA was extracted and mRNA expression was confirmed using qRT-PCR. Expression levels were normalised to NONO mRNA and expressed as fold change relative to vehicle, *n*=3, error bars represent mean ± SEM. **B)** Co-IF analysis of R-loops and UBF in empty-vector or RNAse H-transduced OVCAR8 RAD51C KO cells treated with vehicle or 1 μM CX-5461 for 3 hours. Quantitation of signal intensity was performed using Cell Profiler. *n* =500 cells per treatment condition were examined over three biologically independent experiments. Error bars represent mean ± SD. Statistical analysis was performed using a two-sided Mann-Whitney t test (p-values are shown). **C)** Co-IF analysis of pRPA32 (S33) and UBF in cells treated as in (B). Quantitation of signal intensity using Cell Profiler of n=1100 cells per condition over three independent experiments, error bars represent mean ± SD. Statistical analysis was performed using a two-sided Kolmogorov-Smirnov test (p-values are shown). **D)** Western blot analysis of cells treated with either vehicle, 100nM or 1μM CX-5461 for 24 hours. Representative blots of *n* =3 biologically independent experiments. Blots shown are of samples derived from the same experiment and were processed in parallel. Actin loading controls were processed by re-probing the blots. Full scans of immunoblots are shown in Supplementary Figure 10. **E)** CX-5461 inhibits clonogenic survival of empty-vector and RNAse H-overexpressing OVCAR8 RAD51C KO cells. *n*=9 (three technical replicates were performed over three independent experiments), error bars represent mean ± SEM.

Supplementary Figure 8

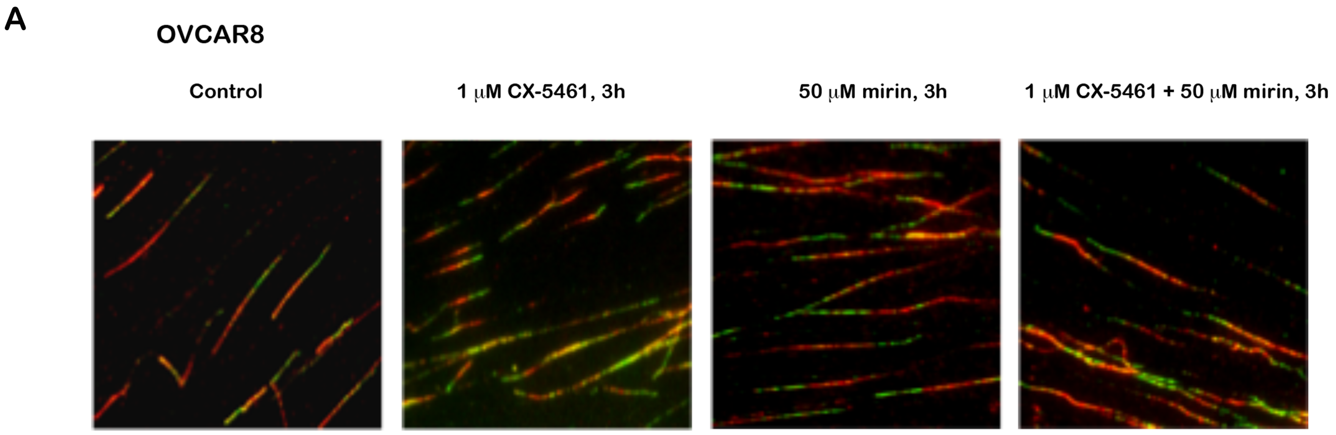

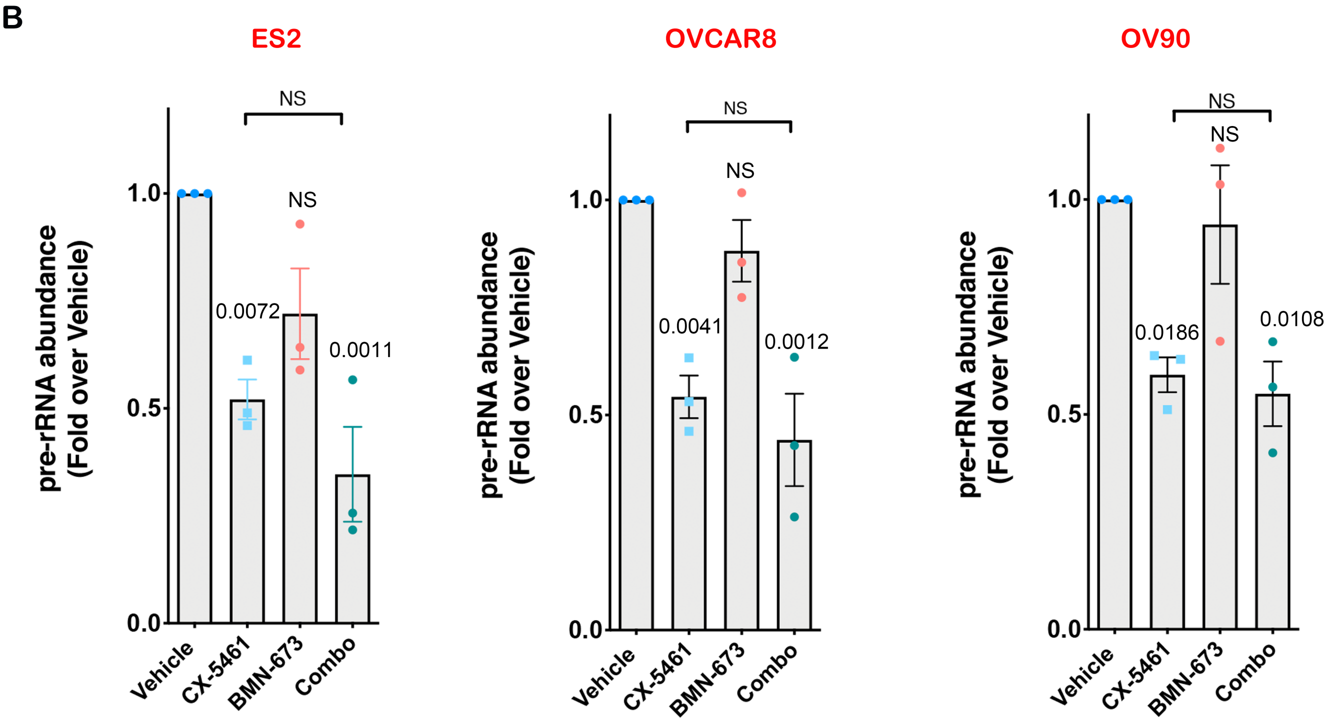

Supplementary Figure 8

A) Representative images of DNA fibre analysis of two independent experiments presented in Figure 6C. OVCAR8 cells were sequentially labelled and either processed or treated with 1 μM CX-5461, 50 mM Mirin or the combination of both drugs for 3 hours. Fibres were processed for DNA fibre analysis; n=150 replication tracks were analysed over two biologically independent experiments. Replication Fork length was calculated based on the length of the IdU tracks measured using ImageJ software.

B) Combined CX-5461 with BMN-673 treatment does not further reduce rDNA transcription rate compared to single agent CX-5461 in OVCA cells. Cells were treated with vehicle, 1μM CX-5461, 100 nM BMN-673 or in combination for 3 hours. RNA was extracted and 47S rRNA precursor levels were determined using primers specific to the 5'ETS. Expression levels were normalised to NONO mRNA and expressed as fold change relative to vehicle (n =3), error bars represent mean ± SEM, statistical analysis was performed using a two-sided one-way ANOVA, Tukey's multiple comparisons test, compared to vehicle samples (adjusted p-values are shown). NS denotes non-significant p-values (greater than 0.05).

Supplementary Figure 9

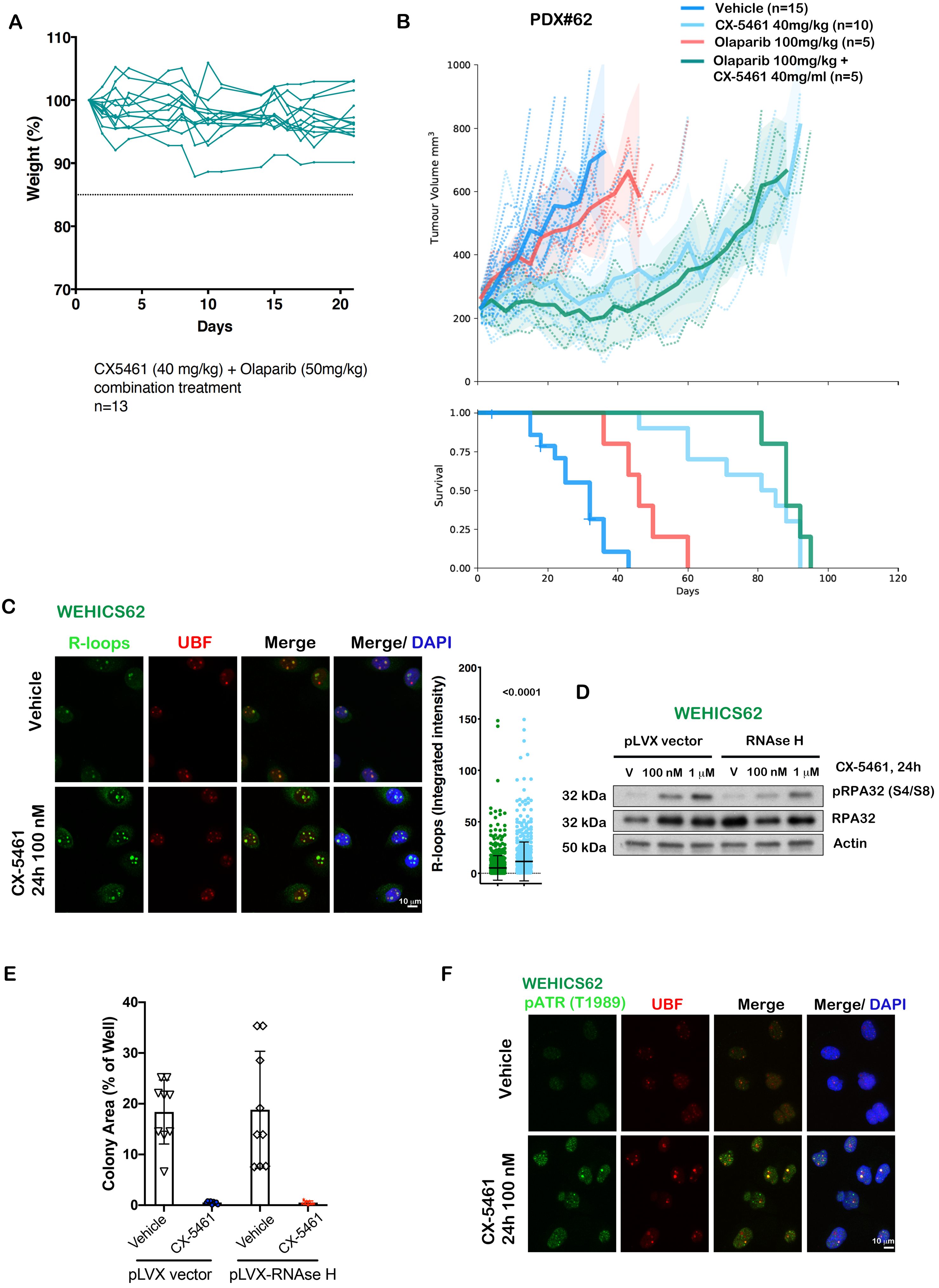

Supplementary Figure 9

A) Effect of CX-5461 and olaparib treatment as described in Figure 8A&B on weight loss during treatment period. **B)** Responses observed in HGSOC PDX#62 with *BRCA1* promoter methylation to CX-5461 and olaparib treatment *in vivo*. Recipient mice bearing the PDX were randomized to treatment with vehicle, 40 mg/kg CX-5461 twice a week, 100 mg/kg olaparib once daily or CX-5461/olaparib combination for 3 weeks. The PDX were harvested at a tumor volume of 700 mm^3. Mean tumor volume (mm^3) (solid lines) ±95% CI (shaded region) and tumour volume of all individual mice (hashed lines) and corresponding Kaplan-Meier survival analysis. Censored events are represented by crosses on Kaplan-Meier plot. n indicates individual mice. **C)** Co-IF analysis of R-loops and UBF in WEHICS62 cells treated with vehicle or 100 nM CX-5461 for 24 hours. Quantitation of signal intensity was performed using Cell Profiler, *n*=500 cells per treatment condition examined over three independent experiments, error bars represent mean ± SD. Statistical analysis was performed using a two-sided Mann-Whitney test (approximate p-values are shown). **D)** Western blot analysis of empty vector or RNAse H overexpressing WEHICS62 cells treated with either vehicle, 100nM or 1μM CX-5461 for 24 hours. Representative blots of *n* =3 biologically independent experiments. Blots shown are of samples derived from the same experiment and were processed in parallel. Actin loading controls were processed by re-probing the blots. Full scans of immunoblots are shown in Supplementary Figure 10. **E)** CX-5461 inhibits clonogenic survival of empty-vector and RNAse H-overexpressing WEHICS62 cells. *n*=9 (three technical replicates were performed over three independent experiments), mean ± SEM. **F)** Co-IF analysis of pATR (T1989) and UBF in cells treated as indicated. Representative images of *n*=3. Quantitation of signal intensity of 500 cells per condition over three independent experiments is presented in Fig 9E.

Supplementary Figure 10

Uncropped immunoblots from Figure 3E

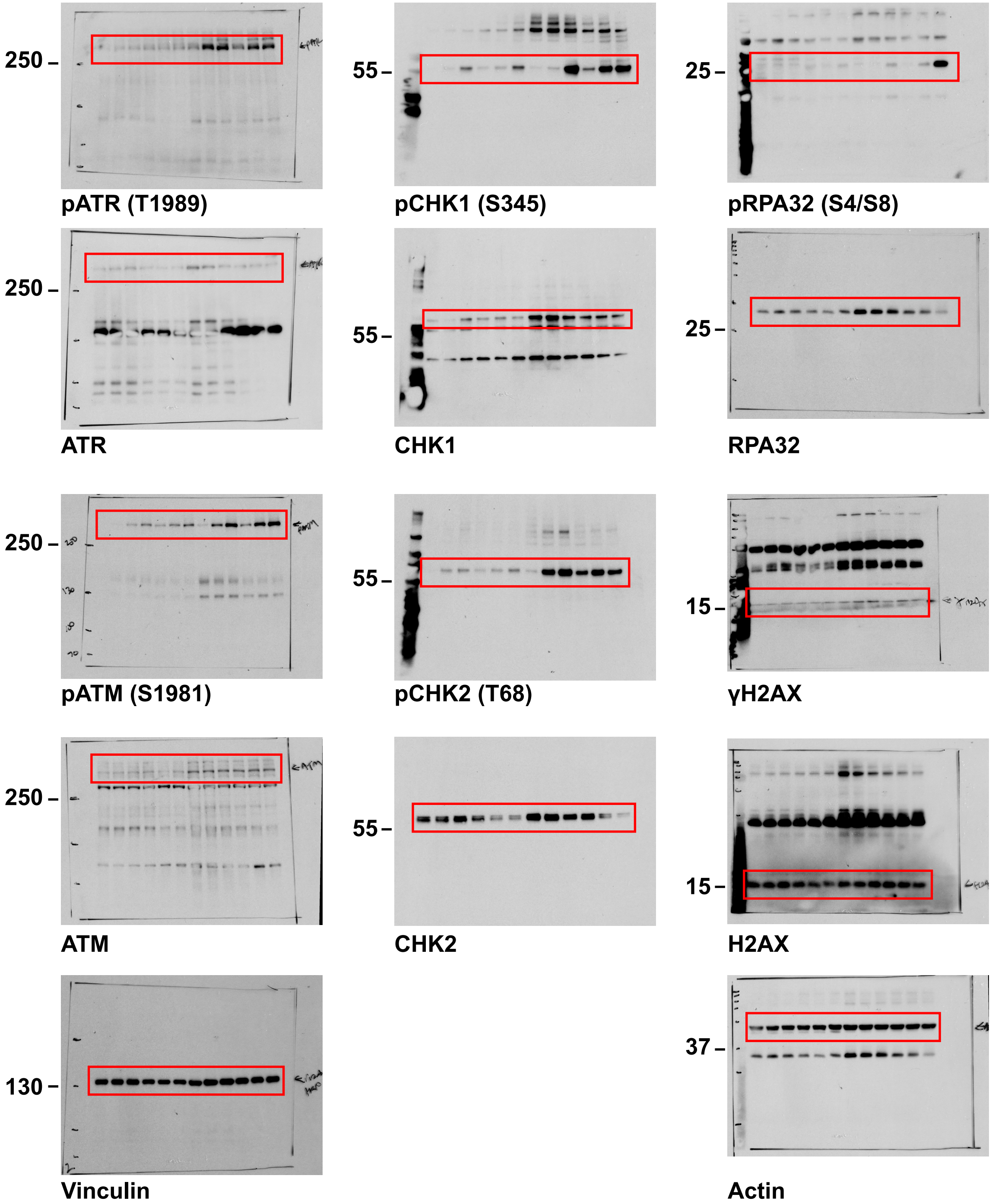

Supplementary Figure 10
Continued

Uncropped immunoblots from Figure 7C

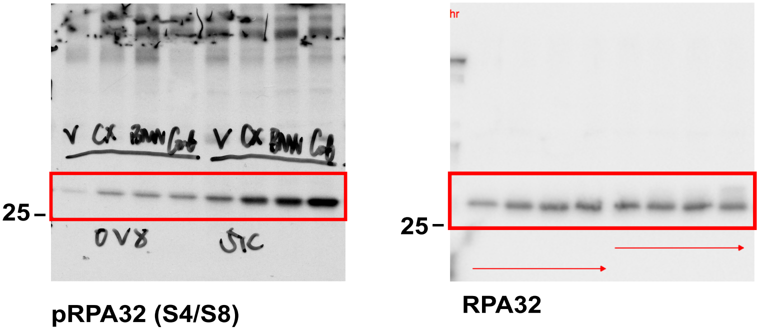

Uncropped immunoblots from Supplementary Figure 4E

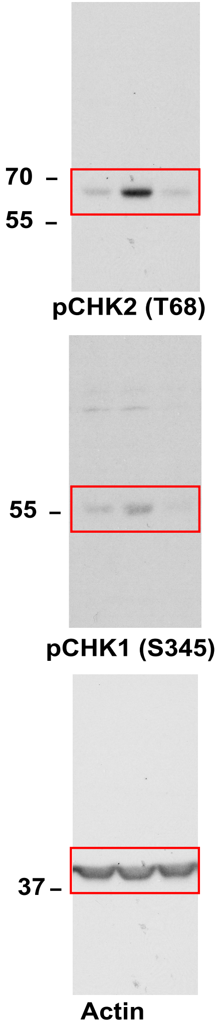

Uncropped immunoblots from Supplementary Figure 7D

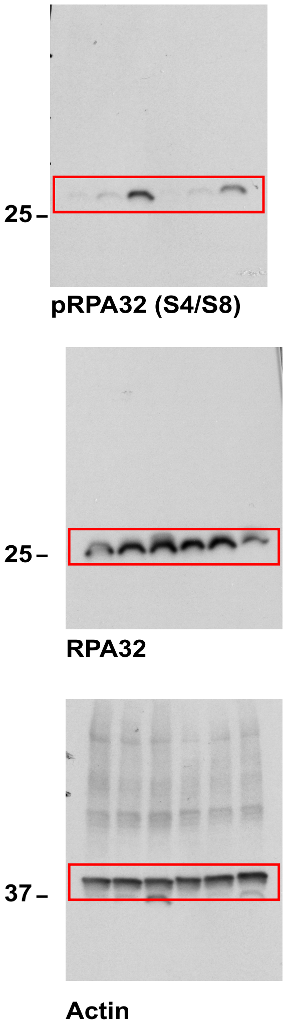

Uncropped immunoblots from Supplementary Figure 9D

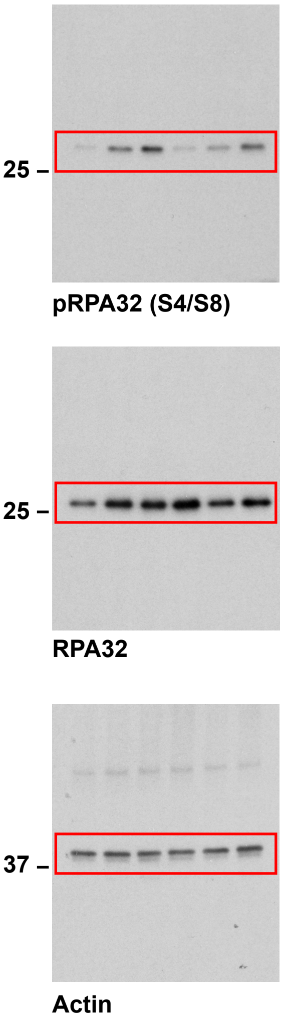

Supplementary Methods

DNA Fibre Assay using Genomic Vision Molecular Combing System: WEHICS62 cells were plated into a 6-well plate at a density of 500,000 cells per well. On the following day, the medium was replaced with culture medium containing 50 µM CldU and incubated at 37°C for 30 minutes. The media was removed, the cells washed three times with phosphate buffered saline (PBS pH 7.4), and new pre-warmed media containing 250µM IdU was added and the cells incubated at 37°C for 30 minutes. After exposure to the second nucleotide analog, cells were washed three times with warm PBS and either processed or treated with pre-warmed media containing vehicle control, 1 µM CX-5461, 2mM Hydroxyurea (Sigma-Aldrich, H8627) (HU) as single agents or in combination for 3 hours at 37°C. Labelled cells were trypsinized, pelleted, washed twice with PBS and resuspended in a pre-warmed solution (50°C) composed of PBS with 0.05% trypsin (no phenol red). Cellular suspensions were then mixed carefully with an equal volume of 1.2% low melt agarose (BioRad, 1613111). This mixture was dispensed into a plug mold (BioRad, 1703713) and allowed to set at 4°C for 1 hour. Solidified plugs were then pushed out of the mold and transferred into 2 ml polypropylene tubes containing 0.5M EDTA pH8, 1% (v/v) Sarkosyl (Sigma-Aldrich, 61743), and proteinase K (Roche Applied Science, 3115828001). The agarose plugs were incubated in this buffer at 50°C overnight. Following incubation, agarose plugs were washed extensively (1M Tris, 0.1M EDTA pH8) and digested with β-agarase (New England Biolabs, M0392S) overnight in 0.5M MES Hydrate (Sigma-Aldrich, M5287) pH5.5 at 42°C. Samples were then poured carefully into a FiberComb reservoir (Genomic Vision, RES-001) and the DNA solution combed onto silanized coverslips (Genomic Vision, COV-002) at a constant speed of 250µm/s, using the Molecular Combing System by Genomic Vision (MCS-001). For easy handling following combing, coverslips were adhered to glass SuperFrost® Plus slides (Menzel Gläser) using cyanoacrylate glue and then baked in an incubator at 65°C for 2 hours to irreversibly crosslink DNA to the surface. Next, samples were immersed in a solution containing 0.5M NaOH and 1M NaCl for 8 minutes at room temperature to denature the combed DNA, before being washed thoroughly with PBS. Coverslips were subsequently dehydrated by incubating them in increasing concentrations of 70%, 90% and 100% of ethanol for 5 minutes each, followed by air drying. To minimise non-specific binding of antibodies, blocking buffer containing 0.1% Triton X-100 and 5% BSA was applied to all slides for 30 minutes at room temperature prior to staining. DNA fibres attached to coverslips were then probed with rat anti-BrdU antibody (1:100, Abcam ab6323) specific to CldU and mouse anti-BrdU antibody (1:50, Becton

Dickinson, 347580) specific to IdU for 1 hour at 37°C in a humidified chamber. Following staining, slides were washed three times with PBS and subsequently incubated with Alexa Fluor 488-conjugated goat anti-rat antibody (Invitrogen,A-11006) and Alexa Fluor 594-labelled donkey anti-mouse antibody (Invitrogen, A-21203) at 1:200 dilutions, for 1 hour at 37°C. Lastly, slides were washed three times in PBS, mounted and visualized using the Nikon C2 confocal microscopy at 40X magnification. Images were taken of 100 fibres per condition. Only high-quality and well separated DNA fibres (not entangled DNA regions) were measured using ImageJ software (1.47v, NIH). The ratio of IdU:CldU tracks in each fibre was calculated and graphed using GraphPad Prism.

Supplementary Table 1. TP53 mutation status assessed by high resolution melting analysis across ovarian cancer cell lines.

Cell Line	Histology	p53 Status	Source	CX-5461 GI50 doses (mean)	SD	N
2008	Endometrioid	WT	Stephen Howell at University of California, San Diego	106.7	19.5	3
59M	Endometrioid	c.577_592del (homo), p.His193LysfsX49	European Collection of Cell Cultures	1386	541.6	3
A2780	Adenocarcinoma SEROUS	WT	European Collection of Cell Cultures	82.33	82.97	3
Caov3	Serous	c.406C>T (homo),p.Gln136Stp	National Cancer Institute	409.3	302.9	3
CH1	SEROUS (Adenocarcinoma)	c.948C>T (het),p.Pro316Pro	Lloyd Kelland at the Institute of Cancer Research, Sutton, UK	73.5	3.536	2
COLO704	Adenocarcinoma	WT	Deutsche Sammlung von Mikroorganismen und Zellkuturen	31.5	14.85	2
Colo720E	Adenocarcinoma	c.1118del (het), p.Lys373ArgfsX49: c.413C>T (het),p.Ala138Val	European Collection of Cell Cultures	413.5	138.7	4
EFO21	Serous	c.370T>C (homo),p.Cys124Ar g	Deutsche Sammlung von Mikroorganismen und Zellkuturen	1835	1082	2
EFO27	Mucinous	c.817C>T (het),p.Arg273Cys	Deutsche Sammlung von Mikroorganismen und Zellkuturen	417	55.49	3
ES2	Serous	c.722C>T Homo),p.Ser241Phe	American Type Culture Collection	181.3	221.7	4
FUOV1	Serous	c.535C>G (homo),p.His179As p	Deutsche Sammlung von Mikroorganismen und Zellkuturen	25.33	26.56	3
IGROV1	Endometriod,serous, clear cell	c.377A>G (het) ,p.Tyr126Cys	National Cancer Institute	422.3	203.7	3
JHOC5	Clear Cell	WT	RIKEN	731	149.8	5
JHOC7	Clear Cell	WT	RIKEN	3267	2003	3
JHOC9	Clear Cell	WT	RIKEN	731	251.8	3
JHOM1	Mucinius	c.637C>T (het),p.Arg213Stp	RIKEN	861.2	1243	5
JHOS3	Serous	c.783-1G>T (homo)	RIKEN	3177	1482	3
KURAMOCHI	Serous	c.841G>T (homo) ,p.Asp281Tyr	Health Science Research Resources Bank	1082	711.5	3
MCAS	Mucinous	WT	Health Science Research Resources Bank	357.6	197.3	5
OAW28	Serous	c.455del (homo), p.Pro152ArgfsX18:	European Collection of Cell Cultures	3850	1431	3
OAW42	Serous	WT	European Collection of Cell Cultures	226.7	25.58	3
OV90	Serous	c.643A>C (Homo),p.Ser215Ar g	American Type Culture Collection	5170	3365	4
OVCA432	Serous	WT	Dr Nuzhat Ahmed, Womens Cancer Research Centre, Royal Women's Hospital, Melbourne	206.7	92.42	3
OVCAR3	Serous	c.743G>A (homo),p.Arg248Gln	National Cancer Institute	12	6.245	3
OVCAR4	Serous	c.388C>G (homo) ,p.Leu130Val	National Cancer Institute	613.3	395.8	3
OVCAR5	Adenocarcinoma	WT	National Cancer Institute	100.5	57.28	2
OVCAR8	Serous	c.376-1G>A (homo)	National Cancer Institute	859	303.8	3
RMGI	Clear Cell	WT	Health Science Research Resources Bank	143	103.2	4
RMGII	Clear Cell	WT	Health Science Research Resources Bank	3370	495	2
SKOV3	Clear Cell	WT	National Cancer Institute	301.3	183.3	3
TOV112D	Endometrioid	c.524G>A (homo),p.Arg175His	American Type Culture Collection	49.67	43.15	3
TOV21G	Clear Cell	WT	American Type Culture Collection	308	140	2

Supplementary Table 2. Antibodies/Reagents			
**Antibody**	**Company**	**Catalogue No.**	**Application, Dilution factor**
Anti-p53 (DO-1)	Santa Cruz	sc-126	WB, 1:1000
Anti-pp53 (S15)	Cell Signaling	9284	WB, 1:500
Anti-pCHK1 (S345) (133D3)	Cell Signaling	2348	WB, 1:1000
Anti-CHK1	Santa Cruz	7898	WB, 1:1000
Anti-pCHK2 (T68) (C13C1)	Cell Signaling	2197	WB, 1:1000
Anti-CHK2	Cell Signaling	2662	WB, 1:1000
Anti-pATM (S1981) [EP1890Y]	Abcam	ab81292	WB, 1:1000
Anti-ATM (2C1)	GeneTex	GTX70103	WB, 1:1000
Anti-pRPA32 S4/S8	Bethyl	A300-245A	WB, 1:1000
Anti-RPA32 (4E4)	Cell Signalling	2208	WB, 1:1000
Anti-pRPA32 S33	Novus Biologicals	NB100-544	IF, 1:100
Anti-Tubulin (B-5-1-2)	Sigma	T5168	WB, 1:5000
Anti-Actin (C4)	MP Biomedicals	08691001	WB, 1:5000
Goat anti-Mouse HRP	Bio-Rad	172-1011	WB, 1:2500
Goat anti-Rabbit HRP	Bio-Rad	170-6515	WB, 1:2500
Rabbit anti-Rat HRP	Dako	P0450	WB, 1:5000
Anti-ATR	GeneTex	GTX128146	WB, 1:1000
Anti-pATR (T1989)	GeneTex	GTX128145	WB, 1:1000; IF, 1;100
Anti-Vinculin (E1E9V)	Cell Signalling	18799	WB, 1:1000
anti-H2AX	Abcam	ab20669	WB, 1:1000
Anti-γH2AX (S139) [EP854(2)Y]	Abcam	ab81299	WB, 1:1000; IF, 1;100
Anti-Rad51	Abcam	ab63801	IF, 1:100

Anti-53BP1 (BP13)	Merck Millipore	MAB3802	IF, 1:100
Anti-DNA G-quadruplex (G4) (1H6)	Merck Millipore	MABE1126	IF, 1:100
Anti-DNA-RNA Hybrid (S9.6)	Kerafast	ENH001	IF, 1:100
Anti UBF (F-9) mouse antibody	Santa Cruz	sc-13125	IF, 1:100
UBF (WT1F) rabbit sera	In-house		IF, 1:200
Goat anti-Rabbit Alexa Fluor 488	Thermo Fisher Scientific	A-11008	IF, 1:100 FACS, 1:800
Donkey anti-Mouse Alexa Fluor 594	Thermo Fisher Scientific	A-21203	IF, 1:100
Goat anti-Mouse Alexa Fluor 488	Thermo Fisher Scientific	A-11001	IF, 1:100
Goat anti-Rabbit Alexa Fluor 594	Thermo Fisher Scientific	A-11012	IF, 1:100
Vectashield	Vectorlabs	H-1000	IF
Vectashield with DAPI	Vectorlabs	H-1200	IF
Anti-BrdU antibody (B44)	BD Biosciences clone (B44)	347580	FACS, 20 μL/μL
Sheep anti-Mouse IgG FITC	MP Biomedicals	0855520	FACS, 1:100
Propidium Iodide	Sigma Aldrich	P4170	FACS
Goat anti-Rat Alexa Fluor 488	Thermo Fisher Scientific	A-11006	Fibre assays, 1:200
Donkey anti-Mouse Alexa Fluor 594	Thermo Fisher Scientific	A-21203	Fibre assays, 1:200
Anti-BrdU antibody Rat monoclonal antibody- specific to CldU	Abcam	ab6326 [BU1/75 (ICR1)	Fibre assays 1:100
Anti-BrdU antibody mouse antibody - specific to IdU	Becton Dickinson	347580	Fibre assays 1:50

Supplementary Table 3: Primer Sequences for quantitative reverse transcription real time-PCR analysis

	Forward	Reverse
47S-rRNA 5’ETS, used in Figure 1C&D and Figure 4E location (+952-1030)	GGCGGTTTGAGTGAGACGAGA	ACGTGCGCTCACCGAGAGCAG
47S-rRNA 5’ETS, used in Supplementary Figure 8B, location (+413-521	GCTCTTCGATCGAGTTGGTGACG	CGGGCGGAGCGAGAAGGAC
RNAse H1	GGTTTCCTGCTGCCAGATTTA	GGCTTGCAGATTTCCTGACAA
Vimentin	AGAGAACTTTGCCGTTGAAGCT	GAAGGTGACGAGCCATTTCC
NONO	CATCAAGGAGGCTCGTGAGAAG	TGGTTGTGCAGCTCTTCCATCC
