# Supplementary material for: CX-5461 activates the DNA damage response and demonstrates therapeutic efficacy in high-grade serous ovarian cancer
Source: Nat Commun. 2020 May 26;11:2641. doi: 10.1038/s41467-020-16393-4 (PMC7251123; doi:10.1038/s41467-020-16393-4)
Supplement: Supplementary file 3 — Description of Additional Supplementary Information [file 41467_2020_16393_MOESM3_ESM.pdf]

Description of Additional Supplementary Files

File Name: Supplementary Data 1
Description: Gene list of HRD signature.

File Name: Supplementary Data 2
Description: Gene list of MYC signature.

File Name: Supplementary Data 3
Description: Gene list of BRCA1-mutated signature.

File Name: Supplementary Data 4
Description: Raw data supporting Figure 1C-E.
