# Supplementary material for: CX-5461 activates the DNA damage response and demonstrates therapeutic efficacy in high-grade serous ovarian cancer
Source: Nat Commun. 2020 May 26;11:2641. doi: 10.1038/s41467-020-16393-4 (PMC7251123; doi:10.1038/s41467-020-16393-4)
Supplement: Supplementary file 4 — Supplementary Data 1 [file 41467_2020_16393_MOESM4_ESM.pdf]

AADAT
ADM
AHSA1
ALDH3B1
ALDH6A1
ALG8
ANLN
ARSD
ASF1B
ASPM
ATP10B
AURKB
BBOX1
BLM
BRCA1
BRI3BP
BTG1
BTG2
C10orf119
C10orf73
C11orf82
C13orf3
C14orf145
C15orf42
C16orf59
C16orf68
C17orf41
C1orf112
C1orf54
C1QTNF6
C20orf19
C20orf82
C4orf34
C5orf38
C6orf48
CBLB
CCDC138
CCDC92
CCNA2
CCNB1
CCNB1IP1
CCNE1
CD68
CDC7
CDCA2
CDCA3
CDCA5
CDCA7
CDCA8
CDKN1C
CENPJ
CHAF1A
CHAF1B
CHEK1

CHRNA5
CKB
CPE
CRIP2
CRYAB
CSE1L
CTSC
CYP4F3
DAPK1
DCC1
DCN
DDEFL1
DDIT3
DDX39
DEPDC1
DHFR
DKFZp762E1312
DLG7
DNA2L
DNMT1
DONSON
DPYSL3
DUT
E2F2
EFHD2
ESCO2
ESPL1
EXO1
FAM134B
FAM43A
FAM81A
FAM83D
FANCI
FBLN1
FDPS
FEN1
FHOD3
FLRT3
FOXO3
FXYD3
GEMIN6
GINS2
GINS3
GINS4
GJB2
GPSM1
HELLS
HIST1H1C
HIST1H2BD
HIST2H2AA3
HIST2H2BE
HLA-E
HMGB3
HSD11B2

HSD17B8
HSPCAL3
HSPE1
IL1R2
INSIG1
KCNB1
KIAA0513
KIF11
KIF14
KIF2C
KLHDC9
KNTC1
KRT6B
LAMB2
LEMD1
LHPP
LMNB2
LOC153222
LOC554223
LOC649679
LOC729843
LOC91431
LOH11CR2A
LRP8
LYRM5
MCM2
MCM3
MCM5
MCM7
METTL3
MLF1IP
MLKL
MME
MOSC1
MRTO4
MSH2
MSH6
MSX1
MT1G
MTBP
NCAPD3
NEIL3
NETO2
NFE2L1
NFIL3
NGFRAP1
NUP205
OIP5
PAQR4
PBK
PCNA
PDSS1
PDXP
PEX11G

PHLDA3
PKMYT1
PLCD1
PLEK2
PLK4
POLA2
POLD1
POLE2
POLQ
POLR3K
PPIL5
PPL
PRC1
PROS1
PRPF38A
RAD54B
RAD54L
RECQL4
RFC3
RFC4
RFC5
RIOK3
RNASEH2A
RRAGD
RRM2
SDCBP2
SERTAD4
SFPQ
SFRS2
SHCBP1
SIDT2
SLC25A10
SLC25A13
SLC45A3
SPC25
SRPK2
ST6GALNAC2
STAT2
SUV39H1
TACC3
TAF5
TERF1
TIGA1
TIMELESS
TINF2
TK1
TMC4
TMEM158
TMEM171
TMEM20
TNFRSF14
TRIP13
TTK
TUBA4A

TUBB2C
TUBB4Q
TXNIP
TYMS
UHRF1
VAMP5
VRK1
WDHD1
WDR79
WIPI1
XPC
YPEL5
ZBTB43
ZNF467
