# Supplementary material for: CX-5461 activates the DNA damage response and demonstrates therapeutic efficacy in high-grade serous ovarian cancer
Source: Nat Commun. 2020 May 26;11:2641. doi: 10.1038/s41467-020-16393-4 (PMC7251123; doi:10.1038/s41467-020-16393-4)
Supplement: Supplementary file 5 — Supplementary Data 2 [file 41467_2020_16393_MOESM5_ESM.pdf]

SLC12A8
DCAF4
C1orf51
RPP40
DUSP2
HSPA6
ZNF667
FAM100A
SLC29A2
ECE2
FAM195A
SLC6A8
DDX10
CCDC78
ALG3
GPATCH4
RABEPK
TFB2M
PMM2
C11orf48
WDR74
IPO4
BID
IMP4
NPM1
GPD1L
METTL1
FARSB
PNPT1
EXOSC5
PLD6
HSPD1
TRMT1
NUFIP1
NCL
TTLL12
MYC
UBIAD1
SCFD2
CIRH1A
LSG1
PTRH2
DDX28
UTP14A
SUPV3L1
C20orf27
PAICS
PHB
C1orf107
CCDC86
DKC1
SRM
DDX18
XPO5

PA2G4
MRTO4
ATIC
NOP56
FARSA
AIMP2
SORD
ANAPC1
CDK4
MON1A
ANGEL1
NOP16
BCS1L
MYBBP1A
CCDC137
GNL3
C19orf48
TMEM97
SLC19A1
RRP9
FLAD1
WDR12
NOLC1
ALDH1B1
NOP2
NLE1
UTP20
TCOF1
PCYOX1L
SFXN4
GEMIN5
C10orf2
HS6ST2
NOL6
HK2
C12orf66
PES1
FLJ10661
DHODH
BEND3
KCNQ5
TAF4B
FAM86C
AGPAT5
FAM86B1
PFKM
RCL1
DKFZp686O24166
PCOLCE2
MCOLN2
SLC6A15
