# Supplementary material for: CX-5461 activates the DNA damage response and demonstrates therapeutic efficacy in high-grade serous ovarian cancer
Source: Nat Commun. 2020 May 26;11:2641. doi: 10.1038/s41467-020-16393-4 (PMC7251123; doi:10.1038/s41467-020-16393-4)
Supplement: Supplementary file 6 — Supplementary Data 3 [file 41467_2020_16393_MOESM6_ESM.pdf]

NCK1
ASF1A
MRE11A
MTF2
USP1
SH2D1A
CEP57
DEK
SFRS11
HMGN4
MYBL2
RAD54L
STMN1
RPA1
TOPBP1
PNN
TTF2
EZH2
TFDP1
SFRS13A
SNRPA
MCM4
RPIA
SMC4
HMMR
LBR
KATNA1
CDC20
CDK1
MSH2
TCERG1
RFC3
CDKN2C
RFC4
DDX39
ILF3
CENPA
MCM2
AURKB
DNMT1
RQCD1
NCAPD2
EXOSC8
SKP2
CAD
UBE2S
FOXM1
DNA2
MCM6
NASP
RAD51AP1
NDC80
MCM5
CDC7

BLM
