# Supplementary material for: CX-5461 activates the DNA damage response and demonstrates therapeutic efficacy in high-grade serous ovarian cancer
Source: Nat Commun. 2020 May 26;11:2641. doi: 10.1038/s41467-020-16393-4 (PMC7251123; doi:10.1038/s41467-020-16393-4)
Supplement: Supplementary file 7 — Supplementary Data 4 [file 41467_2020_16393_MOESM7_ESM.pdf]

## Supplementary Data 4

**(Figure 1C) Baseline rDNA transcription rate measured using quantitative RT-PCR using 5'ETS (+413-521bp) primers. Expression levels were normalised to Vimentin mRNA and expressed as fold change relative to TOV112D cells.**

**n=3 for each cell line**

| | | | |
|---|---|---|---|
| OVCAR3 | 0.70509785 | 0.64691703 | 0.96922698 |
| COLO704 | 0.70749298 | 0.66010888 | 0.67409534 |
| TOV112D | 1 | 1 | 1 |
| A2780 | 1.69017383 | 1.53709553 | 0.67654792 |
| 2008 | 0.77714065 | 0.50059529 | 0.73947696 |
| ES2 | 1.26219068 | 1.0333691 | 0.81822384 |
| IGROV1 | 0.78691188 | 0.51907337 | 0.75324859 |
| OVCAR4 | 0.45482765 | 0.27942998 | 0.61514152 |
| JHOC9 | 0.6184457 | 0.51779098 | 0.57290402 |
| OVCAR8 | 0.27347129 | 0.18820587 | 0.51633561 |
| JHOM1 | 0.68730962 | 0.50919189 | 0.55841017 |
| KURAMOCHI | 0.87712244 | 0.58455337 | 0.64404288 |
| EFO21 | 0.46736582 | 0.64230482 | 0.67478013 |
| RMG2 | 1.1605552 | 0.66430156 | 0.47771473 |
| OV90 | 0.39655481 | 0.40854277 | 0.62007082 |

**Dose-response curves of 47S precursor rRNA levels (Figure 1D) measured by quantitative RT-PCR after 1h treatment. Expression levels were normalised to Vimentin mRNA and expressed as fold change relative to vehicle-treated controls.**

| CX-5461 dose (M) | JHOC9 | | | RMG2 | | | A2780 | | | |
|---|---|---|---|---|---|---|---|---|---|---|
| 0 | 1 | 1 | 1 | 1 | 1 | 1 | 1 | 1 | 1 | 1 |
| 1E-09 | 1.047343 | 1.024169 | 0.8050525 | 0.781811 | 0.717029 | 0.6207976 | 1.21064 | 0.7920837 | 0.6449188 | 1.079156 |
| 0.00000001 | 0.9254161 | 0.90494 | 1.001902 | 1.018812 | 0.9291982 | 0.7913888 | 1.226978 | 1.155955 | 0.5551992 | 1.178455 |
| 0.00000003 | 1.057218 | 1.033826 | 0.8358886 | 0.8882236 | 0.4552283 | 0.4563582 | 1.078808 | 0.6055514 | 0.5353643 | 0.9271011 |
| 0.0000001 | 0.8118653 | 0.7939017 | 0.6944798 | 0.8693585 | 0.414027 | 0.3671815 | 0.7784404 | 0.3051019 | 0.724837 | 1.009559 |
| 0.0000003 | 0.5763462 | 0.5635938 | 0.3840144 | 0.5159234 | 0.1240926 | 0.09886462 | 0.4945741 | 0.2868496 | 0.2847649 | 0.3880067 |
| 0.000001 | 0.3586971 | 0.3507604 | 0.2158092 | 0.216443 | 0.1124152 | 0.1026373 | 0.1883856 | 0.1927899 | 0.2616085 | 0.1661037 |
| 0.000003 | 0.08401752 | 0.08215853 | 0.06114087 | 0.06697337 | 0.02896513 | 0.03341116 | 0.1746497 | 0.0509204 | 0.131555 | 0.1644272 |

| TOV112D | | EFO21 | | | OV90 | | | OVCAR8 | | |
|---|---|---|---|---|---|---|---|---|---|---|
| 1 | 1 | 1 | 1 | 1 | 1 | 1 | 1 | 1 | 1 | 1 |
| 1.012184 | 1.216857 | 1.491813 | 1.313013 | 0.8129221 | 1.355861 | 3.224696 | 2.768376 | 1.162004 | 0.9381102 | 0.8876483 |
| 0.9491521 | 2.075428 | 1.115436 | 1.095261 | 1.79176 | 1.368854 | 2.910089 | 2.340129 | 1.156348 | 0.9594179 | 0.9199377 |
| 0.9065195 | 1.402136 | 1.110225 | 1.691815 | 0.986955 | 1.406067 | 3.107655 | 3.034927 | 0.9999604 | 0.9184855 | 0.8174594 |
| 0.7200699 | 1.012648 | 0.7444266 | 0.9773309 | 0.6680288 | 0.9052224 | 2.42 | 1.65467 | 0.5891215 | 0.5888568 | 0.5183711 |
| 0.2910932 | 0.5089503 | 0.241876 | 0.3103221 | 0.4139799 | 0.5867151 | 0.7303082 | 0.7715623 | 0.367082 | 0.201764 | 0.2506745 |
| 0.2245024 | 0.3329923 | 0.2001955 | 0.2398967 | 0.383922 | 0.3212743 | 0.8100126 | 0.7173818 | 0.1509167 | 0.1089273 | 0.1174649 |
| 0.07869691 | 0.1951878 | 0.1871271 | 0.2673349 | 0.00068669 | 0.3469132 | 0.4984437 | 0.6639284 | 0.09517671 | 0.1072736 | 0.1199586 |

| JHOM1 | | | OVCAR4 | | | COLO704 | | | ES2 | |
|---|---|---|---|---|---|---|---|---|---|---|
| 1 | 1 | 1 | 1 | 1 | 1 | 1 | 1 | 1 | 1 | 1 |
| 0.9272323 | 0.9550329 | 0.9965211 | 0.8150885 | 0.6925736 | 0.924708 | 1.388862 | 1.174811 | 0.9567592 | 1.153746 | 0.9991661 |
| 1.160725 | 1.171864 | 1.100897 | 1.050554 | 0.8552533 | 0.8180767 | 1.58833 | 1.201477 | 0.9746238 | 1.443852 | 0.8594062 |
| 0.8692493 | 1.05138 | 0.8576707 | 1.217718 | 0.9529985 | 1.23408 | 1.144641 | 1.010801 | 0.8795192 | 1.007555 | 0.7337924 |
| 0.6535406 | 0.9881601 | 0.8496936 | 0.542647 | 0.5084217 | 0.4690793 | 1.045877 | 0.7198104 | 0.4937438 | 0.3618693 | 0.4772318 |
| 0.4895552 | 0.5341553 | 0.5546988 | 0.1516427 | 0.126097 | 0.1407958 | 0.5430123 | 0.3940348 | 0.1850573 | 0.1012865 | 0.1960503 |
| 0.2375925 | 0.3036179 | 0.2843019 | 0.1018785 | 0.07958321 | 0.07861684 | 0.19037 | 0.2301882 | 0.1500064 | 0.06643719 | 0.07582373 |
| 0.1492036 | 0.09725322 | 0.07587608 | 0.0973425 | 0.07398467 | 0.1166271 | 0.27581 | 0.2068952 | 0.1579805 | 0.06956791 | 0.08957686 |

| | 2008 | | | Kuramochi | | | IGROV1 | | | |
|---|---|---|---|---|---|---|---|---|---|---|
| 1 | 1 | 1 | 1 | 1 | 1 | 1 | 1 | 1 | 1 | 1 |
| 1.133419 | 1.142952 | 0.9218968 | 0.7659652 | 0.6890829 | 1.454456 | 1.17177 | 0.647665 | 0.956973 | 1.100292 | 1.01273 |
| 1.111042 | 1.25035 | 0.8749439 | 0.5461427 | 0.7271444 | 1.526645 | 1.168948 | 0.8495959 | 0.8934079 | 0.8386626 | 0.7438629 |
| 0.7027034 | 1.218447 | 0.753938 | 0.7017277 | 0.6776185 | 1.072349 | 0.8949839 | 0.4119097 | 0.425926 | 0.6157379 | 0.8635862 |
| 0.4706254 | 0.3765964 | 0.4551122 | 0.3115957 | 0.4642638 | 0.6229261 | 0.643595 | 0.2872059 | 0.539431 | 0.6770015 | 0.4021241 |
| 0.2167591 | 0.3412805 | 0.1927114 | 0.2865324 | 0.2030415 | 0.3707895 | 0.2891547 | 0.05696586 | 0.2581542 | 0.3609257 | 0.3675978 |
| 0.1268788 | 0.1259842 | 0.143429 | 0.2614691 | 0.1283727 | 0.2770877 | 0.2730197 | 0.1020837 | 0.1131854 | 0.1982216 | 0.1745694 |
| 0.2274839 | 0.1693493 | 0.1357278 | 0.1227064 | 0.1269093 | 0.4904453 | 0.286773 | 0.08731644 | 0.1036819 | 0.1487647 | 0.1678949 |

OVCAR3

| | |
|---|---|
| 1 | 1 |
| 0.8327824 | 1.023015 |
| 0.6378638 | 0.9279435 |
| 0.7648521 | 0.9345166 |
| 0.3469949 | 0.6220776 |
| 0.3415337 | 0.4663859 |
| 0.1457704 | 0.2498632 |
| 0.1178687 | 0.2698882 |

IC50 doses **(Figure 1E)** were determined from dose response curves in Figure 1D using GraphPad Prism version 8.

| | CX-5461 | | |
|---|---|---|---|
| | IC50(nM) | SEM | N |
| OVCAR3 | 67.4 | 21.7 | 3 |
| COLO704 | 96.8 | 9 | 3 |
| TOV112D | 134 | 33.6 | 3 |
| A2780 | 63.3 | 35.9 | 3 |
| 2008 | 48.8 | 16.2 | 3 |
| ES2 | 55.1 | 6.2 | 3 |
| IGROV1 | 38.4 | 9.4 | 3 |
| OVCAR4 | 88.5 | 3.5 | 3 |
| JHOC9 | 268.3 | 46.7 | 3 |
| OVCAR8 | 97.6 | 7.5 | 3 |
| JHOM1 | 201.7 | 47.7 | 3 |
| KURAMOCHI | 47.8 | 10.4 | 3 |
| EFO21 | 65.4 | 18.3 | 3 |
| RMG2 | 113.5 | 84.1 | 3 |
| OV90 | 117.3 | 13.8 | 3 |
