# Supplementary material for: CX-5461 activates the DNA damage response and demonstrates therapeutic efficacy in high-grade serous ovarian cancer
Source: Nat Commun. 2020 May 26;11:2641. doi: 10.1038/s41467-020-16393-4 (PMC7251123; doi:10.1038/s41467-020-16393-4)
Supplement: Supplementary file 8 — Reporting Summary [file 41467_2020_16393_MOESM8_ESM.pdf]

nature research

Corresponding author(s): Elaine Sanij

Last updated by author(s): April 11, 2020

Reporting Summary

Nature Research wishes to improve the reproducibility of the work that we publish. This form provides structure for consistency and transparency in reporting. For further information on Nature Research policies, see Authors & Referees and the Editorial Policy Checklist .

Statistics

For all statistical analyses, confirm that the following items are present in the figure legend, table legend, main text, or Methods section.

n/a	Confirmed	
☐	☒	The exact sample size (*n*) for each experimental group/condition, given as a discrete number and unit of measurement
☐	☒	A statement on whether measurements were taken from distinct samples or whether the same sample was measured repeatedly
☐	☒	The statistical test(s) used AND whether they are one- or two-sided<br>*Only common tests should be described solely by name; describe more complex techniques in the Methods section.*
☐	☒	A description of all covariates tested
☐	☒	A description of any assumptions or corrections, such as tests of normality and adjustment for multiple comparisons
☐	☒	A full description of the statistical parameters including central tendency (e.g. means) or other basic estimates (e.g. regression coefficient) AND variation (e.g. standard deviation) or associated estimates of uncertainty (e.g. confidence intervals)
☒	☐	For null hypothesis testing, the test statistic (e.g. *F*, *t*, *r*) with confidence intervals, effect sizes, degrees of freedom and *P* value noted<br>*Give P values as exact values whenever suitable.*
☒	☐	For Bayesian analysis, information on the choice of priors and Markov chain Monte Carlo settings
☒	☐	For hierarchical and complex designs, identification of the appropriate level for tests and full reporting of outcomes
☐	☒	Estimates of effect sizes (e.g. Cohen's *d*, Pearson's *r*), indicating how they were calculated

Our web collection on statistics for biologists contains articles on many of the points above.

Software and code

Policy information about availability of computer code

Data collection

All data in this study was collected via commercial software as described in detail in the appropriate Methods sections. These include a Zeiss ELYRA PS.1 confocal microscope and Olympus BX-61 microscope equipped with a Spot RT camera (model 25.4) and the SPOT Advance (5.6) image acquisition software (Diagnostic Instruments); FACSCanto II (BD Biosciences) and BD FACSAria Fusion 4 (BD Medical Technology) for flow cytometry, Cellomics CX7 for high content imaging, Chemidoc Imaging system (BioRad), iMark™ Microplate Absorbance Reader (BioRad) and IncuCyte ZOOM imaging system (Essen BioScience).

Data analysis

Data analysis in this study is described in detail in the appropriate Methods sections. Publicly available microarray data were analysed via commercial code and/or R-packages, including: the Limma R package (version 3.3.2), GSEA 1000 iterations, the GSVA 53 package (version 1.20.0) in R (version 3.3.2) with details for their specific use described in the Methods section. Other analysis softwares used include GraphPad Prism version 8.0 (GraphPad Software Inc.), Flowlogic software (Version 7.2.1, Inivai Technologies), Cell Profiler version 3.1.9 (Broad Institute), Image J software (1.47v, NIH), SurvivalVolume v1.2, StudyLog software (StudyLog Systems). The source data and the code to reproduce Figures 2A-C, Figure 10 and Supplementary Figure 2 are available in Git-hub as detailed in the data availability section in the article [https://github.com/esanij/CX-5461-sensitivity-signature-in-ovarian-cancer].

For manuscripts utilizing custom algorithms or software that are central to the research but not yet described in published literature, software must be made available to editors/reviewers. We strongly encourage code deposition in a community repository (e.g. GitHub). See the Nature Research guidelines for submitting code & software for further information.

Data

Policy information about availability of data

All manuscripts must include a data availability statement. This statement should provide the following information, where applicable:

- Accession codes, unique identifiers, or web links for publicly available datasets
- A list of figures that have associated raw data
- A description of any restrictions on data availability

The gene expression microarray data of the ovarian cancer cell lines is publicly available in GEO (GSE43765). We have provided a complete data availability statement in the article as suggested. Gene expression data from CCLE and ICGC are publicly available from the original sources [https://portals.broadinstitute.org/ccle/data, https://dcc.icgc.org/]. IC50s from the Genomics of Drug Sensitivity database used for Supplementary Figure 2 are also publicly available from [https://www.cancerrxgene.org, version v17.3]. All the databases/datasets used in the study along with appropriately accessible links and accession numbers in the manuscript under the 'Data availability' section as well as in this reporting summary.

There is no restriction on data availability.

Field-specific reporting

Please select the one below that is the best fit for your research. If you are not sure, read the appropriate sections before making your selection.

☒ Life sciences ☐ Behavioural & social sciences ☐ Ecological, evolutionary & environmental sciences

For a reference copy of the document with all sections, see nature.com/documents/nr-reporting-summary-flat.pdf

Life sciences study design

All studies must disclose on these points even when the disclosure is negative.

Sample size	N/A
Data exclusions	No data were excluded from any analyses.
Replication	All replication attempts were successful. We have used biological (not technical) replicates in this study to demonstrate statistical significance (by a defined methodology). We clearly describe in the manuscript text, relevant Methods sections and Figure Legends precisely how we chose biological replicates (i.e. animal numbers or independent repeats of in vitro experiments). For example, for the western blot assays and proliferation assays, we have shown representative data in the main figure but we have performed at least three independent experiments.
Randomization	Tumour bearing mice were randomly assigned to treatment groups.
Blinding	Section Animal studies (treatment of PDXs) on page 24

Reporting for specific materials, systems and methods

We require information from authors about some types of materials, experimental systems and methods used in many studies. Here, indicate whether each material, system or method listed is relevant to your study. If you are not sure if a list item applies to your research, read the appropriate section before selecting a response.

Materials & experimental systems

n/a	Involved in the study	
☐	☒	Antibodies
☐	☒	Eukaryotic cell lines
☒	☐	Palaeontology
☐	☒	Animals and other organisms
☐	☒	Human research participants
☐	☒	Clinical data

Methods

n/a	Involved in the study	
☒	☐	ChIP-seq
☐	☒	Flow cytometry
☒	☐	MRI-based neuroimaging

Antibodies

Antibodies used	We have reported details for all antibodies used in this study in Supplementary Table 2, with the company and catalog number (and/or clone number) noted as well as antibody application and dilution used.
Validation	All antibodies used in this study have been validated by manufactures for their specific application and previously reported by our group and other labs, which we reference in the article including Quin et al. Oncotarget 2016, Kondrashova et al., Nature Comms 2018 and Hill et al Cancer Discovery 2018.

Eukaryotic cell lines

Policy information about cell lines

Cell line source(s)	Ovarian cancer cell lines were obtained from various sources listed in Supplementary Table 1 or primary citations are referenced to provide this information. FUCCI-labelled cell lines were generated by lentiviral transduction of pCSII-EF-mCherry-hCdt1(30/120) and pCSII-EF-mVenus-hGeminin(1/110) (kindly provided by Dr. Atsushi Miyawaki, RIKEN, Japan).
Authentication	Individuality and the identity of ovarian cell lines were routinely confirmed by a polymerase chain reaction (PCR) based short tandem repeat (STR) analysis using six STR loci.

Mycoplasma contamination	Cell lines were maintained in culture for a maximum of 8-10 weeks. Mycoplasma testing was routinely performed by PCR. Cell lines were only used upon confirmation of mycoplasma negative status.
Commonly misidentified lines (See ICLAC register)	Of the 32 ovarian cell lines used in Figure 1A, the following cell lines are listed as commonly misidentified on the ICLAC register (59M, A2780, Caov3 and CH1). Our STR analysis however confirmed their identity. These cell lines were not used for any other experiments. These 4 cell lines were only used in Figure 1A upon STR authentication of their correct identity.

Animals and other organisms

Policy information about studies involving animals; ARRIVE guidelines recommended for reporting animal research

Laboratory animals	NOD/SCID/IL2Rγnull female mice at 6-8 weeks of age were used. Mouse housing conditions are described on page 24.
Wild animals	No wild animals were used in this study. *Provide details on animals observed in or captured in the field; report species, sex and age where possible. Describe how animals were caught and transported and what happened to captive animals after the study (if killed, explain why and describe method; if released, say where and when) OR state that the study did not involve wild animals.*
Field-collected samples	No field-collected samples were used in this study. *For laboratory work with field-collected samples, describe all relevant parameters such as housing, maintenance, temperature, photoperiod and end-of-experiment protocol OR state that the study did not involve samples collected from the field.*
Ethics oversight	All experiments involving animals were approved by the Walter and Eliza Hall Institute of Medical Research Animal Ethics Committee.

Note that full information on the approval of the study protocol must also be provided in the manuscript.

Human research participants

Policy information about studies involving human research participants

Population characteristics	N/A
Recruitment	Kondrashova et al Nat Comms 2018 had established a cohort of ovarian cancer PDXs from from OC patients enrolled in the Australian Ovarian Cancer Study (http://www.aocstudy.org). In the manuscript we utlilised 2 PDXs based on their response to cisplatin and rucaparib as reported in Kondrashova et al, details in page 14-15.
Ethics oversight	The Australian Ovarian Cancer Study and additional ethics approval was obtained from the Human Research Ethics Committees at the Royal Women's Hospital and the Walter and Eliza Hall Institute, Melbourne. Section "Animal Studies", page 24 Informed consent was obtained from all patients, and all experiments were performed according to the human ethics guidelines.

Note that full information on the approval of the study protocol must also be provided in the manuscript.

Flow Cytometry

Plots

Confirm that:

- [x] The axis labels state the marker and fluorochrome used (e.g. CD4-FITC).
- [x] The axis scales are clearly visible. Include numbers along axes only for bottom left plot of group (a 'group' is an analysis of identical markers).
- [x] All plots are contour plots with outliers or pseudocolor plots.
- [x] A numerical value for number of cells or percentage (with statistics) is provided.

Methodology

Sample preparation	Cell cycle analysis was performed using 5-bromo-2'- deoxyuridine (BrdU) incorporation and propidium iodide staining. Cells were collected, pelleted and fixed in 80% ice-cold ethanol and stored at 4°C until further processing. Detached FUCCI-labelled cells were suspended in PBS and fluorescent protein signals was anlaysed.
Instrument	FACSCanto II (BD Biosciences)
Software	Cell cycle analysis was performed using Flowlogic software (Inivai Technologies) Version 7.2.1
Cell population abundance	Over 10,000 cells were registered per measurment
Gating strategy	The standard gating strategy included FSC/SSC for cell morphology, FSC-A/FSC-H for single cells, FL1-A (FITC)/ FL3-A (Propidium Iodide) We have provided information and a schematic of gating strategy in Supplementary Figure 3D&E.

- [x] Tick this box to confirm that a figure exemplifying the gating strategy is provided in the Supplementary Information.
